# Synaptic withdrawal following nerve injury is influenced by postnatal maturity, muscle‐specific properties, and the presence of underlying pathology in mice

**DOI:** 10.1111/joa.13187

**Published:** 2020-04-20

**Authors:** Alannah J. Mole, Sarah Bell, Alison K. Thomson, Kosala N. Dissanayake, Richard R. Ribchester, Lyndsay M. Murray

**Affiliations:** ^1^ Centre for Discovery Brain Sciences University of Edinburgh Edinburgh UK; ^2^ The Euan MacDonald Centre for Motor Neurone Disease Research Edinburgh UK

**Keywords:** axotomy, developmental regulation, die‐back, differential vulnerability, ex vivo, motor neuron disease, mouse model, neuromuscular disease, neuromuscular junction, peripheral neuropathy, postnatal development, spinal muscular atrophy, synaptic degeneration, Wallerian degeneration

## Abstract

Axonal and synaptic degeneration occur following nerve injury and during disease. Traumatic nerve injury results in rapid fragmentation of the distal axon and loss of synaptic terminals, in a process known as Wallerian degeneration (WD). Identifying and understanding factors that influence the rate of WD is of significant biological and clinical importance, as it will facilitate understanding of the mechanisms of neurodegeneration and identification of novel therapeutic targets. Here, we investigate levels of synaptic loss following nerve injury under a range of conditions, including during postnatal development, in a range of anatomically distinct muscles and in a mouse model of motor neuron disease. By utilising an ex vivo model of nerve injury, we show that synaptic withdrawal is slower during early postnatal development. Significantly more neuromuscular junctions remained fully innervated in the cranial nerve/muscle preparations analysed at P15 than at P25. Furthermore, we demonstrate variability in the level of synaptic withdrawal in response to injury in different muscles, with retraction being slower in abdominal preparations than in cranial muscles across all time points analysed. Importantly, differences between the cranial and thoracoabdominal musculature seen here are not consistent with differences in muscle vulnerability that have been previously reported in mouse models of the childhood motor neuron disease, spinal muscular atrophy (SMA), caused by depletion of survival motor neuron protein (Smn). To further investigate the relationship between synaptic degeneration in SMA and WD, we induced WD in preparations from the *Smn^2B/^* ^−^ mouse model of SMA. In a disease‐resistant muscle (rostral band of levator auris longus), where there is minimal denervation, there was no change in the level of synaptic loss, which suggests that the process of synaptic withdrawal following injury is Smn‐independent. However, in a muscle with ongoing degeneration (transvs. abdominis), the level of synaptic loss significantly increased, with the percentage of denervated endplates increasing by 33% following injury, compared to disease alone. We therefore conclude that the presence of a die‐back can accelerate synaptic loss after injury in *Smn^2B/^* ^−^ mice.

## Graphical abstract

Following traumatic nerve injury, distal synaptic terminals degenerate. Here we show that the rate of degeneration is slower during postnatal development and also varies significantly between anatomically distinct muscles. We further show that the presence of underlying synaptic pathology in a muscle accelerates the rate of synapse loss following injury. This data provides a valuable model system to investigate further the factors which affect synaptic stability and degeneration.

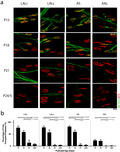

## 1 INTRODUCTION

Motor neurons are vulnerable to degeneration following physical or chemical injury and in a range of neurodegenerative diseases. Damaged axons and synapses degenerate in morphologically distinct ways depending on the nature of the original trauma. Following injury, spontaneous and rapid fragmentation occurs along the length of the injured axon in a characteristic process known as Wallerian degeneration (WD; Waller, 1851; Beirowski *et al.*, 2004, 2005; Conforti *et al.*, 2014). In WD, the cytoskeleton is disassembled and granular degeneration proceeds distal to the injury site (for review see Wang *et al.*, 2012). This is morphologically distinct from degeneration that occurs during peripheral neuropathies, such as amyotrophic lateral sclerosis (ALS) and spinal muscular atrophy (SMA). In some forms of motor neuron disease, axons retrogradely retract away from the synaptic region and are described as ‘dying‐back’ (Cavanagh, 1964). An example of dying‐back pathology can be seen in the childhood motor neuron disease, SMA, where a lack of survival motor neuron (SMN) protein leads to the progressive loss of lower motor neurons (Lefebvre *et al.*, 1995; Rodrigues *et al.*, 1995). Despite morphological distinctions between WD and dying‐back pathology, their mechanistic differences or commonalities remain unclear. Understanding whether different morphologies also relate to different mechanisms would provide a powerful tool to better categorise different types of, neurodegeneration and potentially it will facilitate the identification of novel therapeutic targets.

Recent work has revealed that rates of axonal and synaptic degeneration are influenced by postnatal age*.* Significant differences in the response of neonatal vs. adult mice to peripheral nerve injury and hypoxic insult have been reported (Murray *et al.*, 2011). In young adult mice (P25), tibial nerve lesion results in a loss of motor nerve terminals within the deep lumbrical (DL) muscles, with just 3% of motor endplates remaining innervated after 24 hr (Murray et al., 2011). Contrastingly, in mice aged less than 2 weeks of age, the rate of degeneration is much slower, with 86% of endplates remaining innervated after 24 hr (Murray et al., 2011). Synaptic loss in response to injury therefore appears to be developmentally regulated in the DL muscles. Neonatal, murine neuromuscular junctions (NMJs) also remain structurally intact following exposure to an ex vivo model of hypoxia reperfusion injury, with over 99% of endplates remaining fully occupied in P2 murine transvs. abdominis (TVA), compared with just 4% in adult mice (Murray *et al.*, 2011).

Differential rates of degeneration have been reported between different motor neurons pools in humans and in mouse models of motor neuron disease (Bowerman *et al.*, 2012). Transcriptional profiling of differentially vulnerable motor neurons has identified transcripts and pathways which can modify the rate of degeneration (Hedlund *et al.*, 2010; Brockington *et al.*, 2013; Kaplan *et al.*, 2014; Comley *et al.*, 2015; Murray *et al.*, 2015; Kline *et al.*, 2017; Boyd *et al.*, 2017). Properties that are intrinsic to the motor neuron may therefore regulate the rate of axon degeneration. Understanding the properties of different groups of motor neurons should better inform us of the mechanisms of axon degeneration. Due to the limited numbers of muscle that have been studied in detail, it remains unclear whether there is any variability in the rate of synaptic loss that occurs following injury in different muscles. Determining how different pools of motor neurons respond to injury would provide a useful tool to investigate the factors that regulate WD, and how this relates to other types of axonal and synaptic degeneration.

It remains unclear whether developmental resistance to injury is unique to DL preparations. To investigate this, here we measured synaptic degeneration in cranial and thoracoabdominal musculature following nerve injury. This was achieved by using an ex vivo model of axon injury, where the nerve is transected, and the distal portion along with the musculature it innervates is removed and maintained in oxygenated solutions for 24 hr (Brown *et al.*, 2015; Kline *et al.*, 2019). We have applied this model system to cranial muscle/facial nerve and abdominal muscle/intercostal nerve preparation at different postnatal time points. We show that there is a developmental delay in synaptic withdrawal following injury in all cranial muscles. Interestingly, some muscles (such as adductor auris longus; AAL) showing significantly greater loss than other muscles of the same age (such as levator auris longus; LAL). Furthermore, synaptic degeneration was consistently reduced in the thoracoabdominal muscles compared to the cranial muscles. Together these data suggest that factors intrinsic to the muscle/motor neuron affect the degree of synaptic degeneration following injury. Comparison of the degree of synaptic loss following injury with the degree of synaptic loss in a mouse model of SMA, revealed strikingly different patterns. This suggests that the factors which affect the rate of synaptic degeneration following injury are not the same as those which affect the vulnerability of motor neurons to SMA. Finally, by exposing muscles from the mouse model of SMA to the ex vivo model of nerve injury, we show that the presence of SMA‐induced synaptic pathology significantly increases synaptic loss following injury.

## 2 METHODS

### 2.1 Mouse maintenance

All animal procedures were performed in accordance with the UK Home Office and institutional guidelines. *C57BL/6/J MCos1*, *C57BL/6 Smn* ^2B/2B^ and *Smn* ^+/−^ mice were maintained as groups of less than four (or with parents/littermates) in individually ventilated cages under pathogen‐free conditions, with ad libitum access to food and water, on a 12:12 light/dark cycle within animal facilities at the University of Edinburgh. MCos1 mice were sacrificed between P15‐P25 and *Smn^2B/−^* mice were sacrificed at P18 (disease end‐stage) by an inhalational overdose of anaesthetic (isofluorane). Death was confirmed by exsanguination of the carotid artery. The *Smn* ^2B/−^ mouse is a mouse model of the childhood motor neuron disease, spinal muscular atrophy that exhibits dying‐back pathology. The *Smn2B* allele consists of a three‐nucleotide substitution in the exon enhancer region within exon 7 of the murine *Survival motor neuron* (*Smn)* gene. The resultant mouse has reduced levels of full‐length, endogenous SMN protein which leads to progressive loss of lower motor neurons (DiDonato *et al.*, 2001; Hammond *et al.*, 2010; Bowerman *et al.*, 2012). Onset of disease signs occurs at around P10, and, in our colonies, this model has a lifespan of around 21 days.

### 2.2 Ex vivo model of nerve injury

Cranial and abdominal nerve/muscle explants were dissected as described previously (Murray *et al.*, 2010a, 2014) in Hepes‐buffered saline composed of NaCl (137 mM), KCl (5 mM), CaCl_2_·2H_2_O (2 mM), MgCl_2_·6H_2_O (1 mM), glucose (5.6 mM) and Hepes (5 mM) with pH corrected to 7.4. Nerves (facial or intercostal) were severed at a consistent point, and distal nerve portions with attached musculature were pinned to dental wax and suspended in oxygenated mammalian physiological saline containing: NaCl (120 mM), KCl (5 mM), CaCl_2_·2H_2_O (2 mM), MgCl_2_·6H_2_O (1 mM), NaH_2_PO_4_·2H_2_O (0.4 mM), NaHCO_3_ (23.8 mM), glucose (5.6 mM), gentamicin (50 mg/ml, Thermo Fisher) and kanamycin (100 mg/ml, Thermo Fisher) in a 30‐ml bijou tube. The bijou lid was modified, with two 0.8‐mm needles, one needle clipped short, above the buffer level, to act as a pressure valve. A length of 0.50 mm tubing (VWR) sufficient to reach the bottom of the receptacle was attached to the other needle to provide a constant supply of 95/5% O_2_/CO_2_ to equilibrate the saline, at a perfusion rate of approximately 5 ml/min. The bathing solution was equilibrated for approximately 30 min before adding the sample. The receptacle containing the nerve/muscle explant was then incubated in a 30°C water bath for 24 hr. Temperature was monitored during this period using a digital thermometer (Fisher Traceable Digital Thermometer with stainless steel probe).

### 2.3 Immunohistochemistry

After 24 hr incubation, preparations were fixed immediately in 4% paraformaldehyde (EMS) for 15 min. Muscles of interest were microdissected and permeabilised in 2% Triton‐X‐100‐PBS (Sigma; PBT) for 30 min on a rocking platform at room temperature. Blocking solution (4% bovine serum albumin [BSA]/1% PBT) was applied for 60 min followed by primary antibodies (SV2, 1:100; 2H3, 1:50, both from Developmental Studies Hybridoma Bank) in blocking solution, and left to incubate at 4°C on a rocking platform for 24 hr. Preparations were then washed three times for 5 min with PBS. Secondary antibody (anti‐mouse, Alexa Fluor 488, Stratech, 1:250 in 1×PBS) was added to each well and incubated in the dark at room temperature for a minimum of 4 hr. Muscles were washed three times in 1 × PBS for 5 min, and incubated with tetramethylrhodamine‐conjugated α‐bungarotoxin (TRITC‐BTX; Thermo Fisher, 1:250, 20 min). Muscles were washed and whole‐mounted on glass slides with Mowiol® (Calbiochem) and cover‐slipped. Slides were stored in the dark at 4°C.

### 2.4 Quantification and statistical analyses

**Figure 1:**
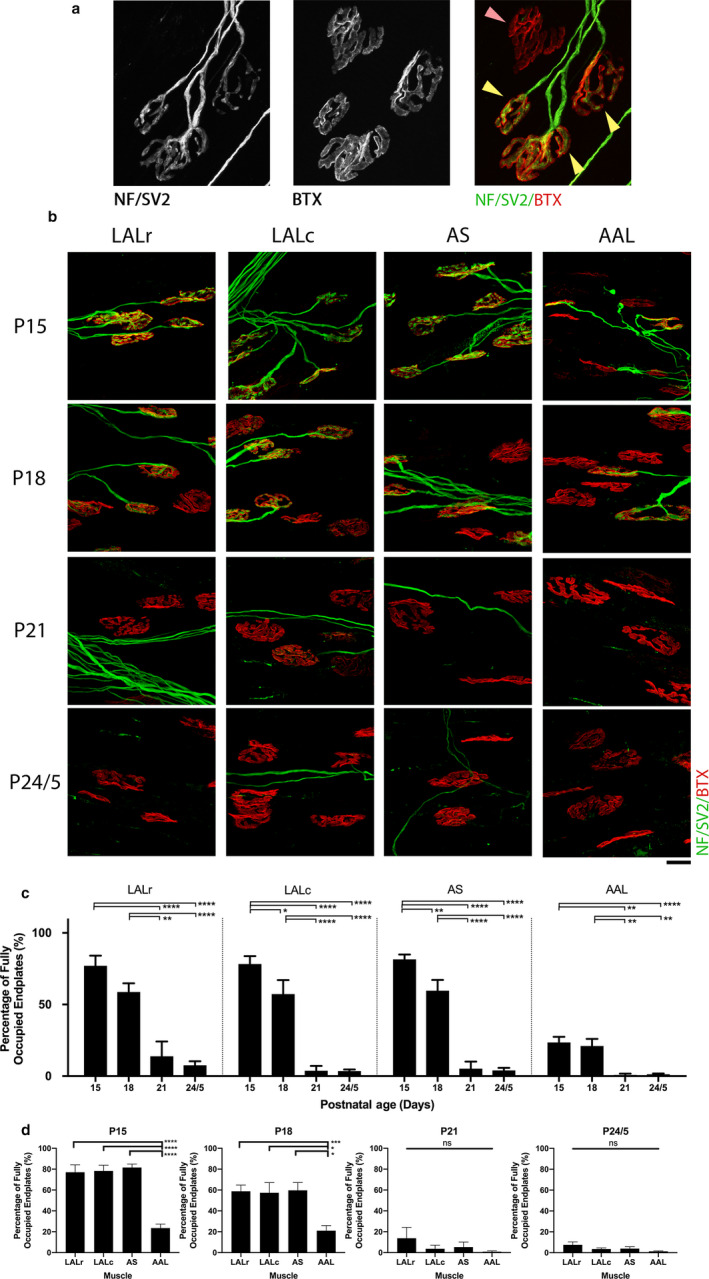
Synaptic stability following injury is developmentally regulated and non‐uniform in cranial muscles of the mouse. (a) Representative confocal micrographs showing example NMJs to exemplify criteria for occupancy quantification. NMJs are labelled with antibodies against neurofilament (NF, green) and synaptic vesicle protein 2 (SV2, green), and α‐bungarotoxin (BTX, red). The yellow arrowheads highlight endplates that would be quantified as ‘full’, whereas the pink arrowhead indicates a vacant endplate, where apposition between the pre‐synaptic terminal and the motor endplate has been lost (see Methods for further detail). (b) Representative confocal micrographs showing NMJs labelled with antibodies against NF (green) and SV2 (green), and BTX (red) from cranial muscles (levator auris longus rostral/caudal band [LALr/c], abductor auris longus [AAL], auricularis superior [AS]) that have been maintained ex vivo at 30°C for 24 hr. Postnatal age refers to the age of the animal at the time of dissection. Scale bar: 20 μm. (c) Bar charts showing quantification of the percentage of fully innervated motor endplates in cranial muscle bands between P15 and P24/5. Note that the percentage of fully occupied endplates decreases as postnatal age increases, supporting the finding that the response to injury is developmentally regulated. (d) Bar charts showing the percentage of fully occupied endplates in cranial muscles grouped by postnatal age at the time of ex vivo nerve injury. Note that there is a significant reduction in the percentage of fully occupied endplates in the AAL at both P15 and P18 time points in comparison with all other muscles. *p < .05, **p < .01, ***p < .001, ****p < .0001; one‐way ANOVA with Tukey’s correction. *n* = 10 at P15 and P24/5; *n* = 18/6 for the LALr/all other muscles at P18; and *n* = 4/5 at P21 for the AAL/all other muscles. Error bars represent mean ± SEM

Quantification of NMJ occupancy was undertaken by fluorescent microscopy at ×40 magnification. The investigator was blinded to the age, genotype and treatment of the muscle. A minimum of two to three fields of view (depending on muscle size and NMJ distribution) and 50 NMJs were sampled per muscle (average of 118 motor endplates per muscle). Fields of view were chosen at random from across the entire muscle using bungarotoxin staining only, to ensure a fair representation of the entire muscle and to reduce the chance of bias for innervated areas. A set of previously optimised and standardly used protocols for NMJ quantification were applied to quantify the percentage of fully occupied endplates (Murray *et al.*, 2008a, 2011, 2013, 2015; Thomson *et al.*, 2012; Sleigh *et al.*, 2014a, 2014b; Brown *et al.*, 2015; Comley *et al.*, 2016; Kline *et al.*, 2017; Courtney *et al.*, 2019; Osman *et al.*, 2019). In brief, fully occupied endplates were defined as complete apposition between the pre‐synaptic terminal and the motor endplate (see Figure 1a for example). This has been expressed as a percentage of the total number of endplates quantified for each muscle, with the remainder of endplates displaying synaptic withdrawal, appearing either only partially innervated (where only part of the AChR cluster is covered by a pre‐synaptic terminal) or vacant (where no pre‐synaptic staining is evident at the AChR cluster).

All morphological analyses were collated into Microsoft excel before exporting to GraphPad prism 8 software for statistical analysis with *n* numbers referring to the number of muscles analysed. Statistical tests are detailed in the legend of the relevant graphs to which they refer. Data were tested for normality using a Shapiro–Wilk test. Data that were normally distributed were tested using one‐way analysis of variance (ANOVA) with post‐hoc Tukey correction. In groups that were not normally distributed, Kruskal–Wallis tests with Dunn’s post‐hoc analysis were applied. All data are presented as mean values ± standard error of the mean (SEM). Differences were considered significant when *p *< .05.

Micrographs are projections of Z‐image stacks collected on a Nikon A1R FLIM confocal microscope and were adjusted for overall image brightness and contrast only using imagej (downloadable from https://imagej.nih.gov/ij/) and Adobe photoshop CS6 software (licence purchased from https://www.adobe.com/uk/products/).

## 3 RESULTS

### 3.1 Synaptic loss following injury is developmentally regulated and non‐uniform in cranial muscles of the mouse

It is unclear whether developmental regulation of synaptic loss in response to injury is unique to the DL muscles or whether developmentally regulated delays in synaptic withdrawal also occurs in other muscle preparations. We therefore examined rates of synaptic withdrawal following injury during the critical time window of P15–P25, using an ex vivo model of nerve injury (see Section 2 for details). Nerve‐muscle preparations comprising facial nerve (CN VII) branches innervating the cranial musculature (comprising rostral and caudal bands of levator auris longus [LALr/LALc, respectively], abductor auris longus [AAL] and auricularis superior [AS]) were isolated and maintained in oxygenated physiological solution at 30°C for 24 hr. We hypothesised that synapse loss would be slower in younger mice and that the level of synaptic loss would differ between different muscles. We tested this by analysing levels of muscle innervation following 24‐hr exposure to the ex vivo model of nerve injury at different postnatal ages.

The percentage of fully innervated or ‘occupied’ endplates remaining in immunostained muscles from P15–P25 mice, 24 hr after ex vivo nerve injury were quantified (Figure 1a–d). Note that all ages refer to the age at dissection. The data show that postnatal age affected the degree of synapse loss in all preparations analysed (Figure 1c). For example, at P15, the LALr remained highly innervated, with 77.05 ± 7.05% (mean ± SEM) of endplates covered by motor nerve terminals. As expected, levels of innervation 24 hr after injury declined with age to 58.75 ± 6.06% innervation in preparations from mice aged P18, to 13.81 ± 10.4% aged P21, and 7.53 ± 2.84% aged P24/5. The LALc and AS muscles followed similar patterns. However, we found significantly more denervated endplates in AAL from the outset, with only 23.56 ± 3.85% innervation remaining at P15. This declined with mouse age to 21.02 ± 4.96% at P18, whereas less than 1.3% innervation remained at P21 and P24/5 (Figure 1d). As both AAL and LAL are homogeneously fast twitch muscles, these differences cannot be attributed to fibre type. This data suggests that there are other intrinsic properties of nerve or muscle that influence the rate of synaptic degeneration during postnatal development.

### 3.2 Levels of synaptic loss are uniformly reduced in thoracoabdominal muscles of the mouse

**Figure 2:**
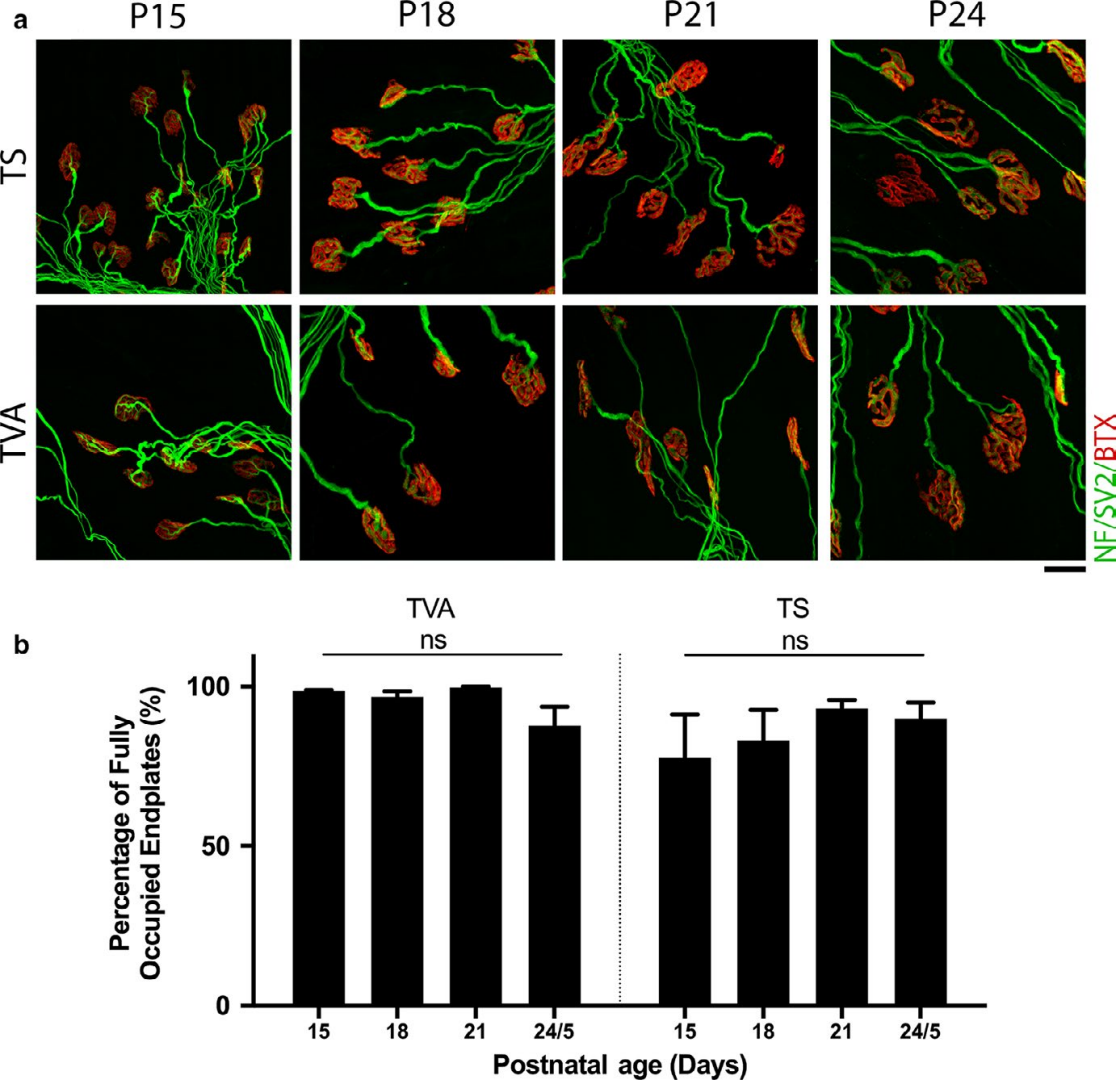
Minimal synaptic loss is observed following injury across all time points analysed in thoracoabdominal musculature. (a) Representative confocal micrographs showing NMJs labelled with antibodies against neurofilament (NF, green) and synaptic vesicle protein 2 (SV2, green), and α‐bungarotoxin (BTX, red) from transvs. abdominis [TVA] and triangularis sterni (TS) muscles which have been maintained ex vivo at 30°C for 24 hr. Postnatal age refers to the age of the animal at the time of dissection. Note that the majority of endplates remain fully occupied at all ages examined. Scale bar: 20 μm. (b) Bar charts showing quantification of the percentage of fully innervated motor endplates after ex vivo nerve injury in the TVA and TS at P15, P18, P21, and P24/5. The percentage of fully occupied endplates remains high across all time points and there is no significant difference within muscles at any time point. ns = not significant, *p* ≥ .09; one‐way ANOVA with Tukey’s correction. TVA, *n* = 6, 18, 4, and 6 muscles, at P15, 18, 21, and 24/5, respectively, and *n* = 6, 6, 6, and 4 in the TS at P15, 18, 21, and 24/5, respectively. Error bars represent mean ± SEM

Data thus far suggest that developmental delays are present in a range of muscles. However, differences between the rates of synaptic withdrawal observed in the AAL at different time points, led us to ask whether there would be similar differences within thoracoabdominal musculature. To investigate the response to injury in these muscles, we examined the TVA and TS muscles 24 hr after ex vivo nerve injury using the same protocol as for cranial muscles. In contrast to cranial preparations, both the TVA and TS showed high levels of NMJ innervation across all time points analysed (Figure 2). There was no significant difference in the percentage of fully occupied endplates at any time point; in all cases, the muscle remained over 77% innervated (one‐way ANOVA with Tukey correction, *p* ≥ .09).

Taken together, the data indicate that there are properties intrinsic to the muscle.., and/or motor neuron, that can influence the extent of synaptic withdrawal in response to injury during the postnatal period.

### 3.3 Patterns of differential syanptic stability following injury contrast with patterns of selective vulnerability in a mouse model of SMA

Variability in the levels of synaptic degeneration, in a given muscle, has previously been reported in mouse models of motor neuron disease. For instance, in the *Smn^2B/−^* mouse model of SMA, the TVA muscle is highly vulnerable and displays high levels of synaptic loss, whereas the LALr and AAL muscles are relatively spared, with little synaptic loss ‘even at late stages of disease’ (Murray *et al.*, 2015). To investigate how synaptic stability differs between disease and injury, we compared levels of innervation remaining in muscles from end‐stage (P18) *Smn^2B/−^* mice in vivo with levels of innervation remaining in homologous muscles from wild‐type animals after ex vivo nerve injury. This provided insight into how synaptic responses differ following these distinct insults.

**Figure 3:**
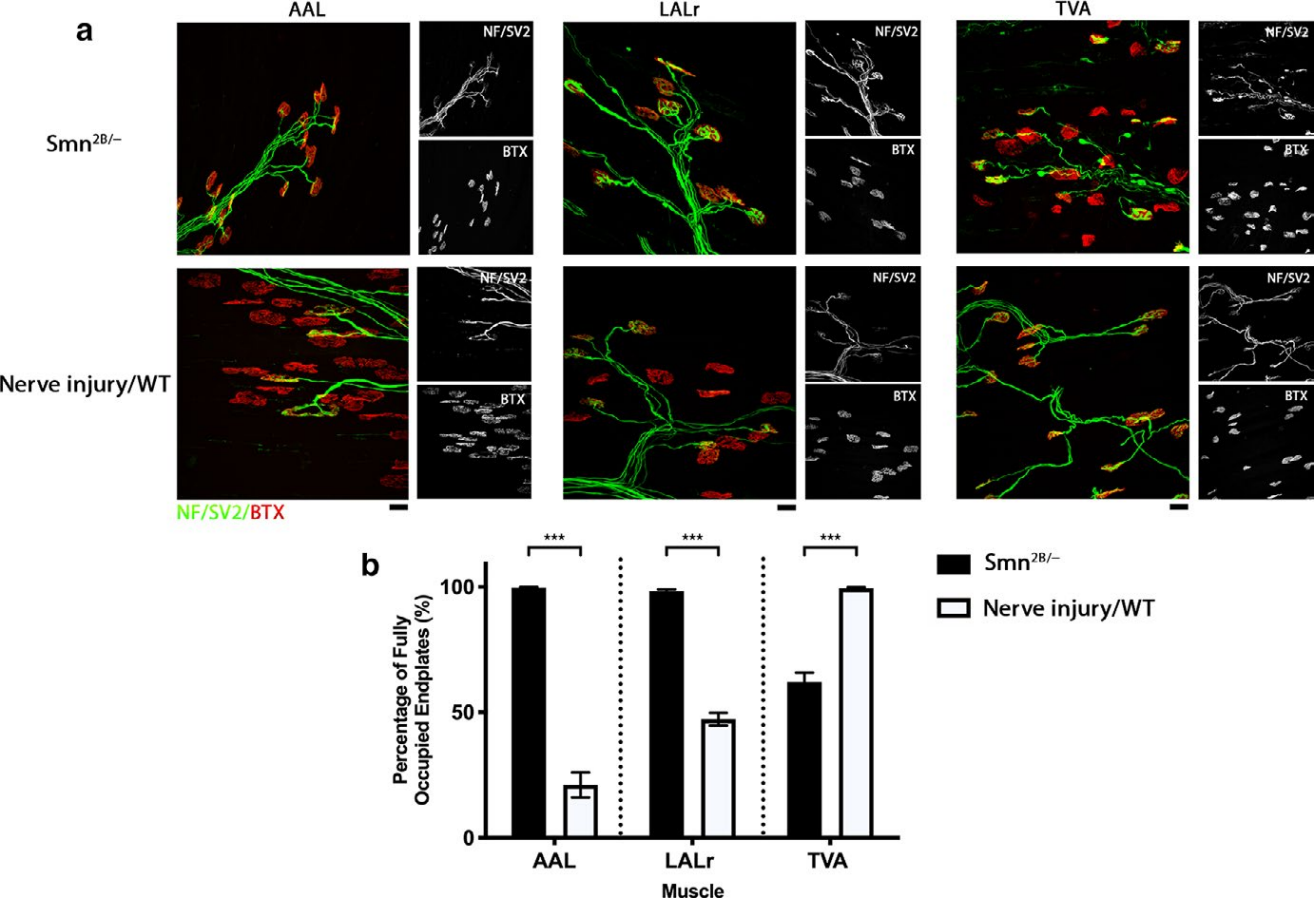
Synaptic stability is different in response to disease or injury conditions. (a) Representative confocal micrographs showing NMJs labelled with antibodies against neurofilament (NF, green) and synaptic vesicle protein 2 (SV2, green), and α‐bungarotoxin (BTX, red) showing innervation of abductor auris longus (AAL), levator auris longus rostral band (LALr), and transvs. abdominis (TVA) under disease conditions from* Smn^2B/−^* mice at P18 and following ex vivo nerve injury in wild‐type animals at P18. Nerve/muscle explants from the ‘nerve injury’ group were maintained ex vivo at 30°C for 24 hr. Scale bar: 20 μm. (b) Bar chart showing quantification of the percentage of fully innervated motor endplates in the AAL, LALr, and TVA after ex vivo nerve injury in WT vs. *Smn^2B/−^* mice. In the *Smn^2B/−^* mouse, the LALr and AAL cranial muscles are spared from degeneration in comparison with the TVA muscle. Following ex vivo* nerve* injury, LALr and AAL show high levels of synapse loss, whereas the TVA appears preserved. ns = not significant, ***p < .001; Mann–Whitney *U*‐test. Note that for the AAL, *n* = 9, 6; for the LALr, *n* = 8, 6; and for the TVA, *n* = 10, 6 muscles for *Smn^2B/−^* and nerve injury/WT groups, respectively. Error bars represent mean ± SEM

In line with previous work undertaken by Murray *et al. *(2015), here the LALr and AAL of *Smn^2B/−^* mice were largely spared from dying‐back neuropathy in vivo, with over 98% of motor endplates remaining fully innervated at P18 (Figure 3). Contrasting this, 24 hr after ex vivo nerve injury in P18 wild‐type mice, the LALr was 47.23 ± 2.58% innervated and the AAL only 21.02 ± 4.96% innervated (Figure 3). These cranial muscles therefore appear to exhibit more extensive synaptic loss following injury than in an SMA mouse model (Mann–Whitney *U*‐test, *p* ≤ .0007). Also in line with descriptions by Murray et al. (2015), in the *Smn^2B/−^* model, the TVA is vulnerable to dying‐back pathology in vivo, with 62.11 ± 3.78% innervation remaining at P18, compared with 99.54 ± 0.29% innervated in wild‐type age‐matched mice 24 hr after ex vivo nerve injury (Figure 3). This suggests that the TVA exhibits less extensive synaptic loss following injury than in an SMA mouse model (Mann‐Whitney *U* test, *p* = .0002). Thus, the factors responsible for determining relative synaptic stability following both nerve injury and SMA, appear to be distinct.

### 3.4 Reduced Smn levels do not influence the incidence of synaptic loss following injury in the LALr cranial muscle of the mouse

We next asked whether a reduction in Smn levels affects synaptic stability after injury. To address this, we isolated and maintained cranial nerve/muscle preparations from wild‐type mice and from *Smn^2B^* ^/−^ mice. In *Smn^2B/^* ^−^ mice, cranial muscles are classified as selectively resistant, in that even at end‐stage of disease, there is no synapse loss evident (cf. Figure 3). Therefore, these muscles have reduced Smn levels but no resultant synaptic loss (Murray *et al.*, 2015). We compared levels of denervation in the LALr under three conditions: LALr from wild type after ex vivo nerve injury; LALr from *Smn^2B/^* ^−^; and LALr from *Smn^2B/^* ^−^ after ex vivo nerve injury.

**Figure 4:**
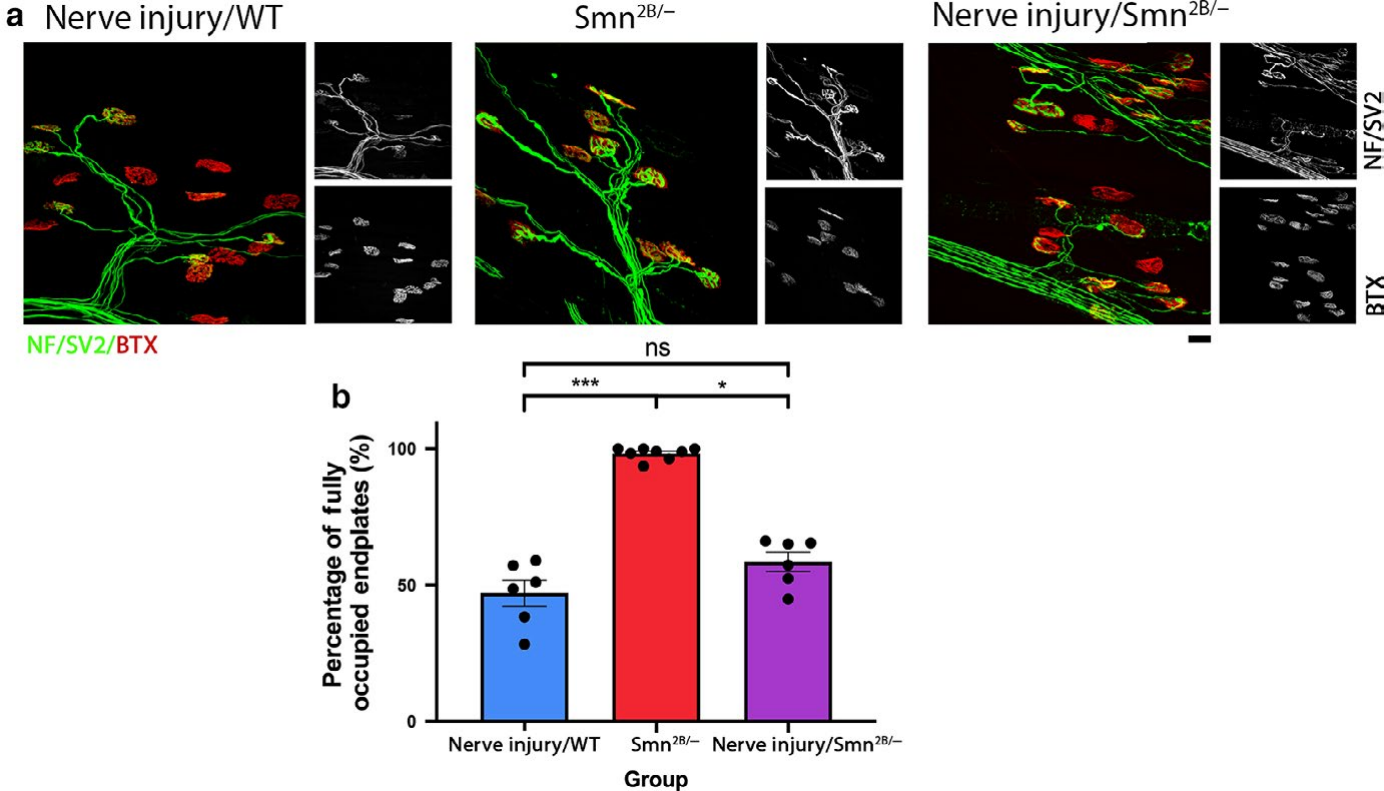
Reduced Smn levels have no effect on the level of synaptic degeneration following injury in the LALr cranial muscle of the mouse. (a) Representative confocal micrographs showing NMJs labelled with antibodies against neurofilament (NF, green) and synaptic vesicle protein 2 (SV2, green), and α‐bungarotoxin (BTX, red) from levator auris longus rostral band (LALr) in wild‐type animals after ex vivo nerve injury (nerve injury*/*WT), in *Smn^2B/−^* mice and in *Smn^2B/−^* mice after ex vivo nerve injury (nerve injury*/Smn^2B/−^*). In nerve injury/WT and nerve injury*/Smn^2B/−^* conditions, LALr muscles were maintained ex vivo at 30°C for 24 hr. Scale bar: 20 μm. (b) Bar charts showing quantification of the percentage of fully occupied endplates in LALr in nerve injury/WT, *Smn^2B/−^*, and nerve injury/*Smn^2B/−^* conditions. The percentage of fully occupied endplates after nerve injury in *Smn^2B/−^* mice is not significantly different than after nerve injury in wild‐type animals. *p < .05, ***p < .001, ns = not significant. For all comparisons with the *Smn^2B/−^* group, Kruskal–Wallis with Dunn’s correction was used to account for non‐normally distributed data; otherwise a one‐way ANOVA with Tukey’s correction was used. Note that *n* = 6, 8, 6 for the nerve injury/WT, *Smn^2B/−^* and nerve injury/*Smn^2B/−^* groups, respectively. Error bars: mean ± SEM

In this disease‐resistant muscle, consistent with previous work by Murray *et al. *(2015), minimal denervation was found in vivo in *Smn^2B/^* ^−^ mice, with over 93% of endplates remaining fully occupied. In wild‐type mice, ex vivo nerve injury‐induced extensive denervation, with 47.12 ± 4.08% of endplates remaining fully occupied. In muscles from *Smn^2B/^* ^−^ mice subject to ex vivo nerve injury, we found that the level of synaptic loss was comparable to wild‐type levels, where 58.55 ± 3.528% of endplates remaining fully occupied (Unpaired *t*‐test with Welch’s correction, *p* = .087; Figure 4). We conclude that in the LALr, where synapse loss due to Smn deficiency is minimal, the synaptic loss caused by nerve injury is not altered. As Smn levels are reduced in this model, despite the lack of NMJ pathology, we conclude that synaptic withdrawal following ex vivo nerve injury is Smn‐independent.

### 3.5 The presence of dying‐back pathology increases the level of synaptic loss following injury in the TVA muscle of the mouse

To investigate the relationship between synaptic loss that occurs in injury vs. disease, we also investigated whether there was any alteration in the level of synaptic loss following injury in the presence of dying‐back pathology. Unlike the LALr, the TVA muscle of the *Smn^2B/^* ^−^ mouse model exhibits high levels of synaptic pathology, including denervation, pre‐synaptic swelling, and post‐synaptic shrinkage at end‐stages of disease (P18). We compared levels of denervation in the TVA under three conditions at P18: TVA from wild type after ex vivo nerve injury; TVA from *Smn^2B/^* ^−^; and TVA from *Smn^2B/^* ^−^ after ex vivo nerve injury.

**Figure 5:**
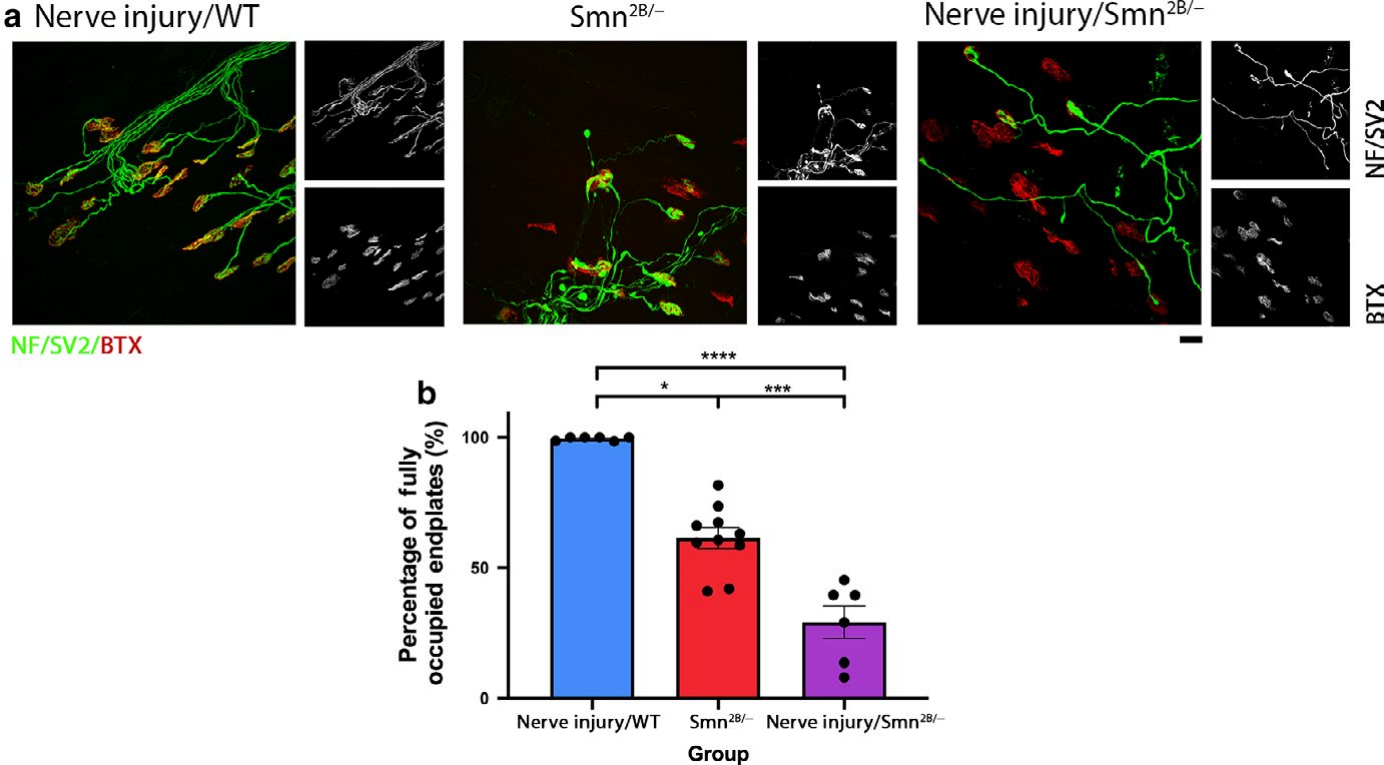
Presence of synaptic pathology in the *Smn^2B/−^* mouse significantly amplifies the level of synaptic loss induced by injury in the TVA muscle. (a) Representative confocal micrographs showing NMJs labelled with antibodies against neurofilament (NF, green) and synaptic vesicle protein 2 (SV2, green), and α‐bungarotoxin (BTX, red) from transvs. abdominis (TVA) at P18 in wild‐type animals after ex vivo nerve injury (nerve injury/WT), in the *Smn^2B/−^* mice, and in *Smn^2B/−^* after ex vivo nerve injury (nerve injury/*Smn^2B/^*) conditions, respectively. In ex vivo conditions, TVA muscles were maintained ex vivo at 30°C for 24 hr. P18 refers to the postnatal age of the animal at the time of dissection. Scale bar: 20 μm. (b) Bar charts showing quantification of the percentage of fully occupied endplates in the TVA in nerve injury/WT, *Smn^2B/−^*, and nerve injury/*Smn^2B/−^* conditions. The percentage of fully occupied endplates following injury in *Smn^2B/−^* mice was significantly increased in comparison with either wild‐type injury or *Smn^2B/−^* conditions in isolation. *p < .05, **p < .01, ****p < .0001. For all comparisons with the nerve injury/WT group, Kruskal–Wallis with Dunn’s correction was used to account for non‐normally distributed data, otherwise a one‐way ANOVA with Tukey’s correction was used. Note that *n* = 6, 10, 6 muscles for the nerve injury/WT, *Smn^2B/−^*, and nerve injury/*Smn^2B/−^* groups, respectively. Error bars: mean ± SEM

Ex vivo nerve injury of the TVA muscle from *Smn^2B/^* ^−^ mice resulted in a significant reduction in the percentage of fully occupied endplates, to just 8.05 ± 6.23% in comparison with wild‐type TVA with ex vivo nerve injury (98.53 ± 0.29% of endplates fully innervated, Kruskal–Wallis with Dunn’s correction, *p* < .0001) and to *Smn^2B/^* ^−^ in vivo (41.11 ± 3.98% of endplates fully innervated, Unpaired *t*‐test with Welch’s correction, *p* = .0018) in isolation (Figure 5). Overall, as we have already established that SMN reduction alone is not sufficient to increase levels of synaptic loss, it follows that the increase in synapse loss observed here is induced by the presence of disease‐induced synaptic pathology.

## 4 DISCUSSION

In this study, we have applied an ex vivo assay to investigate the effect of traumatic nerve injury in a range of muscles that have not been examined in this context previously. We have demonstrated that postnatal maturity influences the synaptic stability following injury in the cranial muscles. We have also shown that levels of synaptic loss are non‐uniform in these muscles, with a significant increase in the rate of synaptic withdrawal in response to injury in the AAL at younger ages when compared with other cranial muscles. We also found that high levels of innervation persisted in the thoracoabdominal muscles after injury across P15–P25. This suggests that muscle and/or motor neuron intrinsic properties affect synaptic stability following injury. Comparison of the patterns of relative synaptic stability following injury to the patterns of synaptic vulnerability in a mouse model of SMA further suggests that the factors which affect synaptic stability following injury are distinct from those in peripheral neuropathies such as SMA. By exposing a muscle from a mouse model of SMA that is resistant to dying‐back pathology with the ex vivo nerve injury assay, we have shown that synaptic loss in response to injury is SMN‐independent. Furthermore, exposure of a muscle with ongoing dying‐back pathology to the ex vivo nerve injury assay has demonstrated that the presence of dying‐back pathology accelerates the rate of synaptic loss in response to injury.

### 4.1 Synaptic stability following injury is governed by properties intrinsic to the muscle and/or motor neuron

We have shown that synaptic withdrawal in response to injury can be influenced by developmental age. Across all four cranial muscles analysed, we observed an increase in the incidence of denervated endplates with increasing postnatal age. These findings are in line with previous work that has shown that following either peripheral nerve injury or hypoxic insult, resistance is gradually lost across the first few weeks of life as the muscle transitions to adult levels of vulnerability (Murray *et al.*, 2011). Furthermore, some muscles, like the AAL, reach adult levels of degeneration at an earlier time point, whereas others, such as the TVA, remain comparatively stable following injury, even at P25 (Murray *et al.*, 2011). This suggests that postnatal maturity as well as properties specific to the muscle and/or motor neuron, are capable of regulating synaptic degeneration following insult. Discerning these mechanisms would be of great value and would contribute significantly to our understanding of the mechanisms regulating synaptic stability and vulnerability to injury and disease.

Indeed, various genetic mutations have been shown to alter the rate of WD in animal models, and have massively increased our understanding of the mechanisms of WD. This includes Wld^s^ and Sterile Alpha and TIR motif‐containing 1 (SARM1) knockout (Lunn *et al.*, 1989; Ribchester *et al.*, 1995; Mack *et al.*, 2001; Wang *et al.*, 2001; Gillingwater *et al.*, 2002, 2004, 2006; Coleman, 2005; Coleman and Freeman, 2010; Gilley and Coleman, 2010; Osterloh *et al.*, 2012; Di Stefano *et al.*, 2015; Gilley *et al.*, 2015)*.* Furthermore, differences in synaptic vulnerability in motor neuron disease have been exploited to investigate the underlying factors that may be responsible making motor neurons vulnerable in motor neuron disease. Transcriptional profiling of differentially vulnerable motor neurons has helped identify transcripts and pathways which can modify the rate of degeneration (Hedlund *et al.*, 2010; Brockington *et al.*, 2013; Kaplan *et al.*, 2014; Comley *et al.*, 2015; Murray *et al.*, 2015; Boyd *et al.*, 2017; Kline *et al.*, 2017). We have recently used this approach to identify alpha‐synuclein and stathmin 1 as phenotypic modifiers of pathology in motor neuron diseases. Importantly, when levels of alpha‐synuclein or stathmin 1 were increased, disease severity was reduced and NMJ pathology ameliorated (Kline *et al.*, 2017; Villalon *et al.*, 2019). Identification of motor neurons that are differentially affected during axon degeneration therefore serves as a useful tool to identify factors that can modify axon degeneration in both disease and injury.

Here we have shown that muscle and/or motor neuron‐specific properties are another factor that can influence the rate of synaptic loss, with responses varying between muscles. This is unlikely to be attributed to muscle fibre type. For example, both LAL and AAL are predominantly fast twitch muscles, but they display a marked difference in synaptic stability following injury (Erzen *et al.*, 2000; Murray *et al.*, 2008a, 2010a, 2010b). In wild‐type rats and in Wld^s^ mice, the rate of synaptic loss has also been shown to be dependent on nerve stump length, with failure of neuromuscular transmission occurring more rapidly when nerve stump length is shorter (Slater, 1966; Ribchester *et al.*, 1995). However, the differences we have reported here cannot be attributed to differences in nerve stump length, as stump length is the same for all of the cranial muscles.

A promising avenue which may explain the differing synaptic stability following injury in different muscles at different postnatal ages, is differences in mitochondrial bioenergetics. In a recent study, we have profiled the nerve and muscle proteome in tibial nerve/lumbrical muscles between P12 and P24, and report a consistent up‐regulation in proteins involved with mitochondrial bioenergetics in both muscle and nerve. Interestingly, this same profile has been observed in the tibialis anterior muscle, and during postnatal development of the heart (Puente *et al.*, 2014; Kim *et al.*, 2019). Importantly, we further showed that inhibition of complex I of the mitochondrial respiratory chain could slow axon degeneration, whereas pharmacological activation of oxidative phosphorylation accelerated synaptic degeneration (Kline *et al.*, 2019). It will now be important to investigate mitochondrial bioenergetics in the range of ages and muscles investigated in this study, to determine whether this can explain the differences in synaptic stability observed. This will help identify factors responsible for conferring rate differences and will provide insight into the mechanisms of synaptic degeneration, and aid in the identification of targets to manipulate the rate of degeneration.

### 4.2 Mechanisms of synaptic loss following injury vs. dying‐back neuropathy

Following traumatic injury in the *Smn^2B^* ^/−^ mouse model of SMA, we assessed the influence on the level of synaptic loss. Differential vulnerability is a known feature in this disease model, therefore we investigated both a die‐back‐resistant (LALr) and die‐back‐vulnerable (TVA) muscle (Murray *et al.*, 2015). As synaptic withdrawal after injury was amplified in the presence of a die‐back, we conclude that the presence of dying‐back pathology can increase synaptic loss in following injury.

Our results are perhaps initially surprising based on previous work where the presence of dying‐back pathology reduced synaptic loss and axon fragmentation following nerve injury. In *Wasted* mice, which display a dying‐back neuromuscular phenotype, translation elongation factor eEF1A2 expression is required to prevent dying‐back pathology at the NMJ, whereas loss of this factor inhibits the initiation and progression of WD (Murray *et al.*, 2008b). Although differing requirements for eEF1A2 expression may represent a mechanistic distinction between injury‐induced degeneration and dying‐back pathology, this does not exclude the possible occurrence of convergence in degenerative pathways further downstream.

Commonalities between WD and dying‐back neuropathies have been reported in animal models of motor neuron disease. For example, *Wld^s^* has been found to be protective in some motor neuron disease and peripheral neuropathy models, suggesting that there may be some convergence between degenerative mechanisms. Similarly, in mice with progressive motor neuronopathy, *Wld^s^* expression also delays dying‐back pathology (Ferri *et al.*, 2003). Delays have also been reported in animal models of Parkinson’s and demyelinating diseases (Conforti *et al.*, 2014). Several similarities have also been highlighted at the cellular level between WD and dying‐back pathology, including poor axonal transport, mitochondrial dysfunction, and an increase in intra‐axonal calcium (for review see Coleman, 2005; Conforti *et al.*, 2014). Our recent proteomic work in mice has identified alterations in mitochondrial bioenergetics that coincide with delays in the change in level of synaptic loss in response to injury during postnatal development in the hindlimb (Kline *et al.*, 2019). Targeting bioenergetic pathways by enhancing mitochondrial bioenergetics has also been found to rescue motor axon defects in a model of SMA (Boyd *et al.*, 2017). Alterations in mitochondrial dynamics may therefore represent a promising, and perhaps uniting factor in WD and dying‐back neuropathy. Overall, the identification of common factors capable of influencing the rate of neurodegeneration under different physiological conditions could aid in the identification of targets that are common across multiple diseases.

## CONFLICT OF INTEREST

The authors have no conflicts of interest to declare.

## AUTHOR CONTRIBUTIONS

A. J. Mole: Design of experiments in Figures 1, 2, 3, 4, 5, acquisition, analysis, and interpretation of data for Figures 1, 2, 3, 4, 5; drafting of manuscript. S. Bell: Acquisition and processing of data for Figures 4 and 5, approval of manuscript. A .K. Thomson: Design of experiments for Figures 1 and 2, optimisation of experimental systems, critical reading and approval of manuscript. K. N. Dissanayake: Concept and design of Figures 1 and 2, approval of manuscript. R. R. Ribchester: Concept and design, critical reading and approval of manuscript. L. M. Murray: Concept and design of study, critical revision of the manuscript and approval of the manuscript.

## Data Availability

The raw data that support the findings of this study are available from the corresponding author (L.M.M.) upon reasonable request.
